# A Human/Murine Chimeric Fab Antibody Neutralizes Anthrax Lethal Toxin *In Vitro*

**DOI:** 10.1155/2013/475809

**Published:** 2013-06-05

**Authors:** Guipeng Ding, Ximin Chen, Jin Zhu, Nicholas S. Duesbery, Xunjia Cheng, Brian Cao

**Affiliations:** ^1^Department of Pathology, Nanjing Medical University, 140 Hanzhong Road, Nanjing 210029, China; ^2^Huadong Medical Institute of Biotechniques, 293 East Zhongshan Road, Nanjing 210002, China; ^3^Laboratory of Cancer and Developmental Cell Biology, Van Andel Research Institute, 333 Bostwick Avenue, Grand Rapids, MI 49503, USA; ^4^Department of Medical Microbiology and Parasitology, Shanghai Medical College of Fudan University, 138 Yixueyuan Road, Shanghai 200032, China; ^5^Laboratory of Antibody Technology, Van Andel Research Institute, 333 Bostwick Avenue, Grand Rapids, MI 49503, USA

## Abstract

Human anthrax infection caused by exposure to *Bacillus anthracis* cannot always be treated by antibiotics. This is mostly because of the effect of the remaining anthrax toxin in the body. Lethal factor (LF) is a component of lethal toxin (LeTx), which is the major virulence of anthrax toxin. A murine IgG monoclonal antibody (mAb) against LF with blocking activity (coded LF8) was produced in a previous study. In this report, a human/murine chimeric Fab mAb (coded LF8-Fab) was developed from LF8 by inserting murine variable regions into human constant regions using antibody engineering to reduce the incompatibility of the murine antibody for human use. The LF8-Fab expressed in *Escherichia coli* could specifically identify LF with an affinity of 3.46 × 10^7^ L/mol and could neutralize LeTx with an EC_50_ of 85 **μ**g/mL. Even after LeTx challenge at various time points, the LF8-Fab demonstrated protection of J774A.1 cells *in vitro*. The results suggest that the LF8-Fab might be further characterized and potentially be used for clinical applications against anthrax infection.

## 1. Introduction

Anthrax infection is caused by *Bacillus anthracis* which primarily affects livestock but can spread to humans [1]. It is known that anthrax spores have the potential use as a weapon of bioterrorism. The anthrax attacks of 2001 heightened awareness concerning the treatment of anthrax exposure [2]. One of the current clinical treatments for anthrax is to use antibiotics which are effective but limited [3]. This is mainly because of the effect of the remaining anthrax toxin in the body, which cannot be eliminated by antibiotics. Anthrax toxin consists of three protein components: protective antigen (PA), lethal factor (LF), and edema factor (EF). PA combining with LF or EF constitutes lethal toxin (LeTx) or edema toxin (EdTx), respectively [4]. The 83 kDa form of PA (PA83) binds either of two known receptors on the surface of mammalian cells: anthrax toxin receptor 1 (ATXR1)/tumor endothelial marker 8 (TEM8) or anthrax toxin receptor 2 (ATXR2)/capillary morphogenesis protein 2 (CMG2) [5]. Then, PA83 is cleaved by a furin-like protease, producing PA20 and PA63. The latter oligomerizes to a heptamer and forms a pre-pore to bind LF and/or EF. The complex is internalized into cells by receptor-mediated endocytosis, and LF and/or EF are released to cytosol under acid conditions [6]. LF is the major virulent factor which is responsible for shock and death. LF is a zinc-dependent protease which can cleave several members of mitogen-activated protein kinase kinase (MAPKK) family causing lysis of macrophages [7]. In addition, LF offers *Bacillus anthracis* an efficient mechanism to evade the host immune responses by inhibiting interferon regulatory factor 3 (IRF3) activation by lipopolysaccharide and subsequent cytokine production through bacterial membrane components [8]. EF is a calcium-calmodulin-dependent adenylate cyclase which causes local edema [9].

Recent studies of antitoxin treatments have focused on three aspects: vaccines [10], monoclonal antibodies (mAbs), and other inhibitors, such as dominant-negative mutants of PA [11], soluble receptors [12], and noncatalytic domains of LF and EF [13]. Many neutralizing mAbs against PA have been developed and utilized in clinical trials [14], as PA shares the common part of LeTx and EdTx. However, the neutralization effect may become invalid against mutant strains of *Bacillus anthracis* [15]. Hence, EF and LF mAbs are alternative options to be used alone or in combination with PA mAb [16]. Murine mAbs may have some limitations to be used in humans directly because of the human anti-mouse antibody (HAMA) response [17]. It is necessary to develop mAbs with low immunogenicity including human, humanized, and chimeric mAbs. Human mAbs are generated by technologies of phage display library, transgenic mouse, EBV immortalized human B cell, and human-human hybridoma [18]. Humanized and chimeric mAbs, produced by genetic engineering, have the original target specificity of the murine precursor. Compared to the time-consuming and laborious mutations in development of humanized mAb, chimeric mAb is prepared by recombining of whole murine variable regions, not only CDRs, with human constant regions. Furthermore, in contrast to the repeating administration of the mAb against tumor, the dosage of the anti-infective mAb is not so frequent. Sometimes only a single dose is necessary before or after the exposure to the microorganism [19]. In this situation, chimeric mAb may have as fewer side effects as humanized and human mAbs.

In a previous study, we reported the production of a neutralizing murine mAb (coded LF8) against LF that blocks LeTx formation [20]. In this study, we develop a human/murine chimeric Fab mAb (coded LF8-Fab) which was generated by antibody engineering using LF8 variable regions combined with human constant regions. The LF8-Fab could bind LF specifically and protect J774A.1 cells against LeTx challenge *in vitro* under prophylactic and postexposure conditions. Our results suggest that this chimeric LF8-Fab mAb might be further characterized and potentially be used for clinical treatment of anthrax infection.

## 2. Materials and Methods

### 2.1. Murine LF8 and LeTx

 Murine mAb against anthrax lethal factor (LF8) was developed and purified in our lab, as described previously [20]. Briefly, BALB/c mice were immunized with purified LF protein, and spleen cells were fused with P3X63AF8/653 myeloma cells using standard protocol. The LF8 was screened by ELISA, immune precipitation, Western blotting, and gel mobility-shifting assay. This murine mAb could inhibit LeTx both *in vitro* and* in vivo*. Purified LeTx (PA and LF) was produced in Dr. Nick Duesbery's lab at Van Andel Research Institute [21].

### 2.2. Construction of Expression Vector

Vectors pComb3XSS and pComb3XTT were kindly provided by the Barbas laboratory (Scripps Research Institute, USA). Total RNA was extracted from the LF8 hybridoma cells by the TRIzol reagent, and cDNA was synthesized using SuperScript II Reverse Transcriptase according to the manufacturer's protocols. The prokaryotic vector was constructed by cloning LF8-Fab into pComb3XSS following three rounds of PCR amplification as described previously [22]. First, murine variable regions of the heavy chain (V_H_) and the light chain (V_L_) were amplified by PCR using cDNA of LF8 as template. GAPDH severed as the internal control of RT-PCR using the primers (Forward: 5′-CCCTTCATTGACCTCAAC-3′ and Backward: TTCACACCCATCACAAAC) [23]. Human constant regions of both the heavy chain domain 1 (C_H_1) and the light chain (C_L_) were amplified by PCR using vector pComb3XTT as the template. Second, equimolar quantities of V_H_ and C_H_1 PCR products were used in the overlap PCR to create the heavy chain Fd (Fd), while equimolar quantities of V_L_ and C_L_ PCR products were used to create the light chain. Third, equimolar quantities of Fd and light chain PCR products were used in the overlap PCR to create the Fab DNA. Then, LF8-Fab DNA was cloned into pComb3XSS following single digestion of restriction endonuclease *Sfi* I. The recombinant vector pComb3X/LF8-Fab was sequenced using an ABI 3700-capillary electrophoresis DNA sequencer. Sequences were further analyzed using the VBASE2 database (http://www.vbase2.org/).

### 2.3. Expression and Purification of Human/Murine Chimeric LF8-Fab

The recombinant vector pComb3X/LF8-Fab was transformed into competent *E. coli* HB2151. The LF8-Fab expression was induced by 1 mM isopropyl-*β*-D-thiogalactoside (IPTG) overnight at 30°C. Individual clones were identified by Western blotting using goat antihuman IgG (Fab-specific) and IRDye 800CW donkey antigoat IgG in an Odyssey infrared image system (LI-COR Biosciences, Lincoln, NE). Soluble LF8-Fab in the cell lysate was purified by immobilized metal affinity chromatography (IMAC) using a 5 mL His-trap HP column, followed by ion exchange chromatography (IEC) using a 5 mL Q sepharose column as described previously [24]. The purity of the LF8-Fab was examined by SDS-PAGE (10%) with coomassie blue staining.

### 2.4. Affinity Calculation of the LF8-Fab

The affinity of the LF8-Fab was calculated by noncompetitive ELISA [25]. Ninety-six-well EIA plates were coated overnight at 4°C with LF at two different concentrations, 4 *μ*g/mL and 2 *μ*g/mL. After the plate was blocked, serial concentrations of the LF8-Fab were added (4 replicated wells for each concentration) as the primary antibody. The alkaline phosphatase- (AP-) conjugated antihuman Fab severed as the secondary antibody. The absorbance was read at 405 nm after color development. The concentration of the LF8-Fab and the absorbance at 405 nm were plotted to two hyperbolic curves by GraphPad Prism software version 5.0 (GraphPad Software, Inc., La Jolla, CA, USA). Affinity constant (*K*_ aff _) was calculated using SPSS statistical software version 13.0 (SPSS Inc., Chicago, IL, USA) by equation *K*_ aff_ = 1/(2[Ab′]t − [Ab]t), where [Ab′]t was the free mAb concentration at the OD_50_ of 2 *μ*g/mL coated antigen, while [Ab]t was the free mAb concentration at the OD_50_ of 4 *μ*g/mL coated antigen.

### 2.5. *In Vitro* LeTx Neutralization Assay

 The LeTx *in vitro* neutralization assay was performed as described previously [26]. Murine macrophage J774A.1 cells, cultured in Dulbecco's Modified Eagle Medium (DMEM) with 10% FBS, were seeded in 96-well plates (2 × 10^4^ cells in 100 *μ*L medium per well), 12 h prior to the assay. The LF8-Fab was serially diluted and mixed with LeTx (0.5 *μ*g/mL of PA plus 0.1 *μ*g/mL of LF). The mixture was applied to cells (3 replicated wells for each dilution). Untreated cells and cells with only LeTx acted as controls. Plate was incubated for 3 h at 37°C. Cell viability was determined using a CellTiter 96 AQ_ueous_ assay. The concentration of the LF8-Fab and the percentage of viability were plotted to a hyperbolic curve by GraphPad Prism software. SPSS statistical software was used to calculate the 50% effective concentration (EC_50_). 

### 2.6. *In Vitro* LeTx Challenge Study

 To determine the protection effect of the LF8-Fab under prophylactic and postexposure conditions, cells were treated with either LeTx prior to the LF8-Fab or the converse at various time points (–120, –60, –30, –15, –5, 0, +5, +15, +30, +60, +75, +90, +105, and +120 min). Minus (–) means mAb treatment prior to LeTx, while plus (+) means LeTx prior to mAb treatment. The percentage of cell viability was calculated in the same way as above.

## 3. Results

### 3.1. Construction of Expression Vector

**Figure 1:**
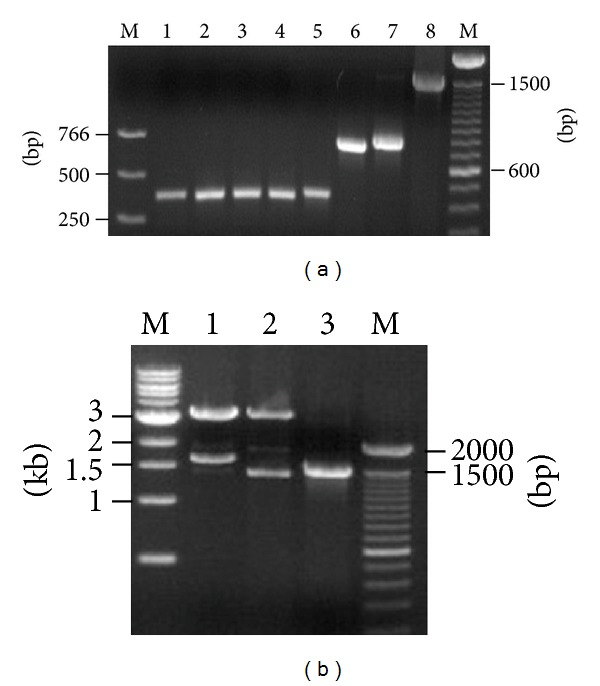
Construction of expression vector. (a) PCR products from three-round amplification. M, DNA marker; lane 1, GAPDH as internal control; lane 2, V_H_; lane 3, V_L_; lane 4, C_H_1; lane 5, C_L_; lane 6, heavy chain Fd; lane 7, light chain; lane 8, Fab. (b) Recombinant vector pComb3X/LF8-Fab recut by *Sfi *I. M, DNA marker; lane 1, pComb3XSS cut by *Sfi *I; lane 2, pComb3X/LF8-Fab recut by *Sfi *I; lane 3, PCR product of Fab.

**Figure 2:**
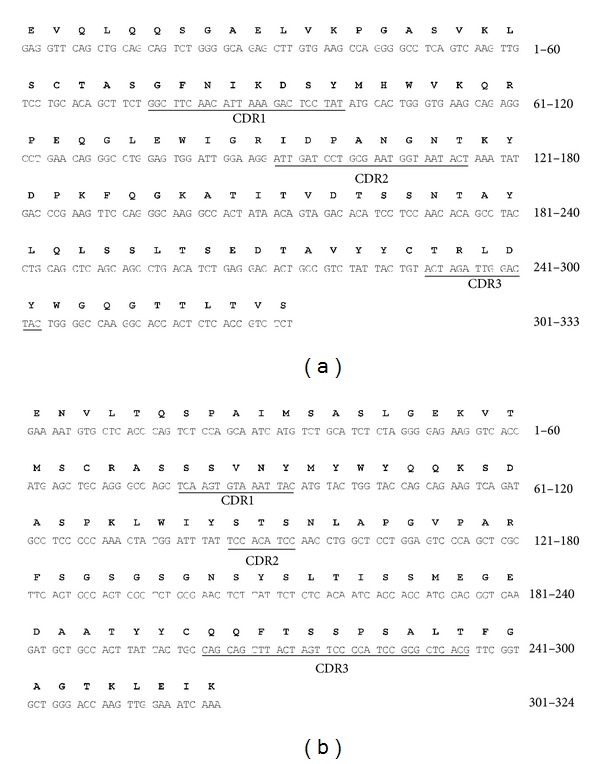
Nucleotide and deduced amino acid sequences of V_H_ and V_L_. The CDRs are underlined based on the analysis of VBASE2 database. (a) Nucleotide sequence of V_H_ and deduced amino acid sequence of V_H_. (b) Nucleotide sequence of V_L_ and deduced amino acid sequence of V_L_.

Total RNA was extracted from the LF8 hybridoma cells, and cDNA was synthesized. As expected, murine V_H_, V_L_, and human C_H_1 products were about 350 bp, while human C_L_ product was about 400 bp. GAPDH severing as the internal control of RT-PCR was also amplified. Then, chimeric heavy chain Fd (about 750 bp), light chain (about 800 bp), and Fab (about 1500 bp) products were consequently achieved (Figure 1(a)). The recombinant vector pComb3X/LF8-Fab was constructed successfully according to sequencing analysis and could be recut by *Sfi *I (Figure 1(b)), indicating the integrity of the vector. The complementary determining region (CDR) and framework region (FR) of V_H_ and V_L_ were determined by VBASE2 database (Figure 2). The V_H_ sequence was one member of the Igh-V15 VH15 family, while the V_L_ sequence belonged to IGKV4/5 subgroup.

### 3.2. Expression and Purification of Human/Murine Chimeric LF8-Fab

**Figure 3:**
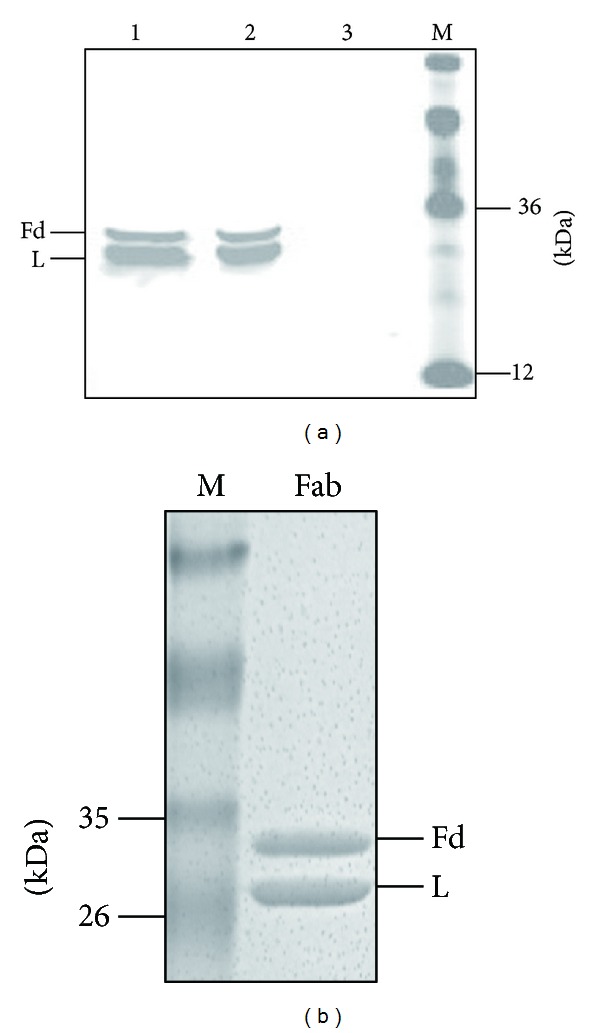
Expression and purification of the LF8-Fab. (a) A goat antihuman Fab and IRDye 800CW donkey antigoat IgG were used to detect Fab expression in Western blotting. Both heavy chain Fd (Fd) and light chain (L) were expressed. Lane 1, supernatant of sonicated lysate; lane 2, pellet of sonicated lysate; lane 3, lysate of untranfected *E. coli* HB2151 as negative control; lane 4, protein marker. (b) Coomassie blue staining showed that heavy chain Fd (Fd) and light chain (L) of the LF8-Fab were equally expressed after the purification of IMAC and IEC.

Recombinant vector pComb3X/LF8-Fab was transfected into competent *E. coli* HB2151. The LF8-Fab expression was induced by 1 mM IPTG overnight at 30°C and then identified by Western blotting in the Odyssey infrared image system. Both heavy chain Fd and light chain were expressed as the expected sizes (Figure 3(a)). The theoretical pI was 7.92, calculated by the Compute pI/Mw tool at ExPASy (http://web.expasy.org/compute_pi/). The optimized IEC eluent was start buffer plus 150 mM NaCl. SDS-PAGE (10%) followed by Coomassie Blue staining showed that heavy chain Fd and light chain were equally expressed, and the purity was above 95% (Figure 3(b)). The output level was about 1 mg purified protein from 1 L *E. coli* culture by BCA protein assay.

### 3.3. Affinity Calculation of the LF8-Fab

**Figure 4:**
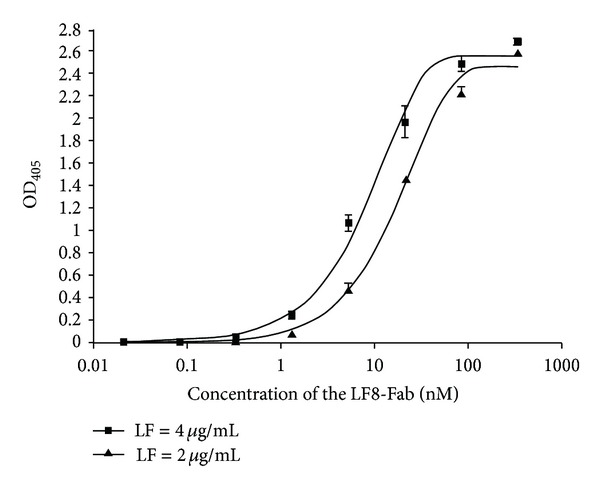
Affinity calculation of the LF8-Fab. Affinity was measured by noncompetitive ELISA. The concentration of the LF8-Fab and the absorbance at 405 nm were plotted to two hyperbolic curves using GraphPad Prism software. The LF8-Fab could identify LF in a dose-dependent manner with an affinity constant of 3.46 × 10^7^ L/mol according to the calculation of SPSS statistical software.

The concentration of the LF8-Fab and the absorbance at 405 nm were plotted to two hyperbolic curves using GraphPad Prism software (Figure 4). The LF8-Fab could identify LF (either at 4 *μ*g/mL or 2 *μ*g/mL) in a dose-dependent manner. Using SPSS statistical software, [Ab′]t was 20.5 nM and [Ab]t was 12.1 nM. According to the equation *K*_ aff_ = 1/(2[Ab′]t − [Ab]t), the *K*_ aff_ of the LF8-Fab was 3.46 × 10^7^ L/mol.

### 3.4. *In Vitro* LeTx Neutralization Assay

**Figure 5:**
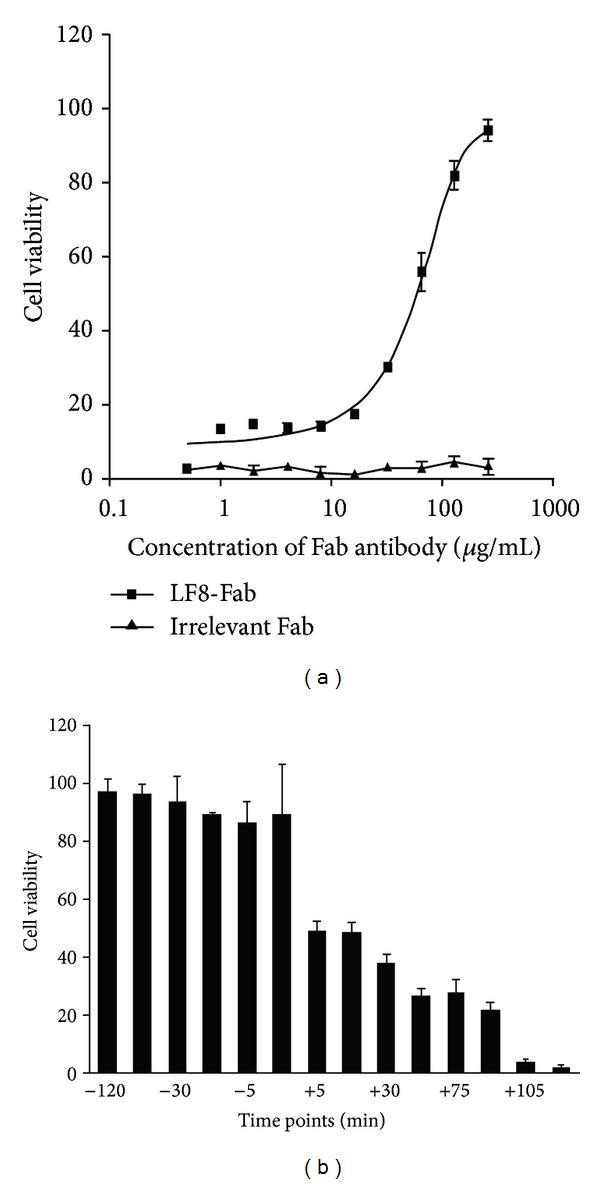
*In vitro* protection of J774A.1 cells against LeTx challenge. (a) *In vitro* neutralization assay showed that LF8-Fab could protect cells against LeTx in a dose-dependent manner, with an EC_50_ of 85 *μ*g/mL according to calculation of SPSS statistical software. The percentage of viability was demonstrated by GraphPad Prism software. (b) *In vitro *LeTx challenge study to test neutralization effect of LF8-Fab before or after LeTx exposure of J774A.1 cells at different time points. Minus (−) means LF8-Fab prior to LeTx, while plus (+) means LeTx prior to LF8-Fab. LF8-Fab could completely neutralize LeTx and protect cells as long as the treatment was prior to LeTx addition. As for the converse situation, protection effect declined in a time-dependent manner.

 The *in vitro* neutralization assay showed that the LF8-Fab could protect cells against LeTx in a dose-dependent manner and could offer 100% protection at a concentration of 5 *μ*M (Figure 5(a)). In contrast, the irrelevant Fab could not protect cells in the presence of LeTx. The EC_50_ of the LF8-Fab was 85 *μ*g/mL, according to the calculation of SPSS statistical software.

### 3.5. *In Vitro* LeTx Challenge Study

In order to determine the protection effect under prophylactic and postexposure conditions, the LF8-Fab was chosen at a concentration of 100% protection (5 *μ*M). LeTx and the LF8-Fab were added to cells at different time points. The results of cell viability indicate that the LF8-Fab could completely neutralize LeTx and protect cells if the mAb treatment was prior to LeTx addition. As for the converse situation when LeTx challenge was prior to the mAb treatment, protection effect declined in a time-dependent manner and had 37.8% protection at +30 min and 26.8% protection at +60 min (Figure 5(b)).

## 4. Discussion

In the present study, we have produced a human/mouse chimeric anti-LF Fab (LF8-Fab) in *Escherichia coli* which could specifically identify LF with an affinity of 3.46 × 10^7^ L/mol and could neutralize LeTx *in vitro* with an EC_50_ of 85 *μ*g/mL. Even after LeTx challenge prior to the mAb treatment, the LF8-Fab demonstrated protection of J774A.1 cells *in vitro*.

Some of anti-LF mAbs (5/9) listed in Table 1 are murine ones. The others include human, chimpanzee, and chimpanzee/human chimeric mAbs. Only one is Fab, while the rest mAbs are all IgGs. Chimeric mAb, like LF8-Fab, keeps a balance between murine mAb which has high affinity and human mAb which has low immunogenicity. Undoubtedly, human mAbs are the most desirable source for clinical application in human body, while molecular modifications of murine mAbs, including the chimeric and humanized mAbs, can reduce the immunogenicity and can retain the similar affinity and stability of murine mAbs as well. Compared to humanized mAb, generation of chimeric one is relatively time and labor saving. Chimeric mAb with two isoforms, Fab and IgG, has equivalent therapeutic effect and less side effect as humanized mAb, especially in the treatment of infection diseases. Fab mAb, consisting of light chain and heavy chain Fd, lacks Fc region which is not necessary for antibody binding. Moreover, Fc region may bind to Fc receptors (FcRs) on the surface of certain cells, including leukocytes, epithelial cells, endothelial cells, and hepatocytes [32]. Thus, Fc region may attenuate the specificity of IgG mAb and increase the dosage of antibody. Fab mAb has the advantage of eliminating nonspecific binding between Fc region and FcRs. Moreover, Fab mAb may penetrate tissues more efficiently, as it has smaller size than IgG mAb (only 1/3 the size of IgG mAb) [33].

VBASE2 is used to analyze sequences of V_H_ and V_L_ of LF8-Fab. This is an integrative database of germ-line variable genes from the immunoglobulin loci of human and mouse, while all variable gene sequences are extracted from the EMBL-Bank [34]. It is often used to analyze variable regions of antibody sequences with intact FR4 information. According to this database, CDRs and FRs of V_H_ and V_L_ were determined. The V_H_ sequence was a member of the IGH15 family, while the V_L_ sequence belonged to IGKV4/5 subgroup. Sequences were also examined in the V-QUEST tool at IMGT database (http://www.imgt.org/), and similar results were achieved. The only differentia was that the V_H_ sequence belonged to the IGH14 family by IMGT. This might be caused by the criterion of different databases. The pComb3X vectors (GenBank accession number AF268281) are phagemids for phage display and antibody expression [35]. In this study, pComb3XTT vector was employed as PCR template to amplify human C_H_1 and C_L_. The other vector, pComb3XSS, was used for Fab expression. The light chain and heavy chain Fd were expressed, respectively, and assembled in the periplasm of bacterium, and soluble Fab mAb was obtained. A number of techniques have used to purify Fab mAb, including antigen affinity, IMAC, IEC, protein L affinity, gel filtration, and so on [36]. As vector pComb3XSS has the His6 tag, so IMAC was carried out first. However, the relatively low purity of LF8-Fab of IMAC (about 60%) was mainly caused by the endogenous histidine residues in *E. coli* HB2151. So, it was necessary to make a secondary purification of IEC by Q sepharose column. According to the preliminary experiment of small scale, the optimized IEC eluent was start buffer plus 150 mM NaCl. This was consistent with the calculation by the Compute pI/Mw tool at ExPASy. With the combination of IMAC and IEC, the purity rose to 95% and could be used in further experiments.

Several methods have been established to measure affinity of mAbs, such as surface plasmon resonance (SPR) [37] and quartz crystal microbalance (QCM) [38]. Here we utilized a simple while reliable method based on noncompetitive ELISA, which utilizes the dose-response curve to calculate an affinity constant. This method is based upon the Law of Mass Action, using total antibody concentration added per well rather than the bound-to-free antigen ratio. It compares the OD_50_ of two sigmoid curves of antibody serial dilutions on a plate coated with the same antigen at two different concentrations. It is generally believed that the Fab (and other monovalents) fragment displays a relatively low affinity than the divalent constructs, such as the F (ab′)_2_ and the full-length immunoglobulin [39]. Compared to the affinity of the LF8 (data not shown), LF8-Fab revealed a moderate affinity against LF.

In *in vitro* assay, the molar ratio of PA and LF consisting of LeTx is the key to LeTx challenge. According to other studies [40] and our preliminary test, 0.5 *μ*g/mL of PA to 0.1 *μ*g/mL of LF is an appropriate ratio of LeTx. Under these concentrations, cell viability of J774A.1 cells dropped to zero in less than 2 h. Hence, employing LeTx 120 min prior to mAb treatment was the last time point under postexposure condition. Neutralization activities *in vitro* demonstrated that the LF8-Fab could protect J774A.1 cells well against LeTx challenge. When mixed with LeTx, LF8-Fab could protect cells against LeTx in a dose-dependent manner and offer 100% protection at a concentration of 5 *μ*M. And then this concentration (5 *μ*M) of the LF8-Fab was used to determine the protection effect under prophylactic and postexposure conditions. LeTx and the LF8-Fab were added to cells at different time points. As expected, the LF8-Fab could completely neutralize LeTx and protect J774A.1 cells if the mAb treatment was prior to LeTx challenge. On the reverse situation when the mAb treatment was posterior to exposure to LeTx, the LF8-Fab could partly retrieve the cells in a time-dependent manner. There was about 37.8% protection at +30 min and 26.8% protection at +60 min after exposure.

Further study will focus on several aspects of this mAb. First, the ability of the LF8-Fab to protect mice against LeTx challenge will be evaluated under both prophylactic and postexposure conditions. Second, the affinity needs to be improved by affinity maturation. Third, this mAb will be characterized in detail (i.e., specificity, toxicity studies, autoantigen testing, etc.). Last, epitope mapping and structure-function analysis of the murine LF8 mAb have been performed. Generally, it is believed that conversion from murine mAb to chimeric one may not change the interaction of antigen and antibody. However, the LF8-Fab lacks Fc region, compared to the LF8. So, mAb epitope analysis of the LF8-Fab is still worth investigating. The epitope mapping of Fab-LF interaction will bring out a better understanding of the neutralization mechanism of the LF8-Fab.

In summary, we report herein the development of a human/murine chimeric Fab mAb, the LF8-Fab, to reduce murine immunogenicity. The LF8-Fab can identify LF specifically with moderate affinity and can neutralize LeTx and protect the macrophage cells *in vitro*. Thus, we believe that LF8-Fab might be further characterized (i.e., specificity, toxicity studies, autoantigen testing, etc.) and potentially be used alone or in combination with other neutralizing mAbs for medical therapy of anthrax infection.

## Figures and Tables

**Table 1:** List of neutralizing mAbs against anthrax LF.

Authors	Source	Isoform	Reference
Little et al. (1990)	Murine	IgG1	[27]
Zhao et al. (2003)	Murine	IgG	[20]
Lim et al. (2005)	Murine	IgG1	[8]
Albrecht et al. (2007)	Human	IgG1	[28]
Staats et al. (2007)	Murine	IgG1	[29]
Chen et al. (2009)	Chimpanzee	Fab	[15]
Chen et al. (2009)	Chimpanzee/human	IgG1	[15]
Kulshreshtha and Bhatnagar (2011)	Murine	IgG2b	[30]
vor dem Esche et al. (2011)	Human	IgG1	[31]

LF: lethal factor.
